# Buprenorphine-Precipitated Withdrawal Among Hospitalized Patients Using Fentanyl

**DOI:** 10.1001/jamanetworkopen.2024.35895

**Published:** 2024-09-27

**Authors:** Ashish P. Thakrar, Paul J. Christine, Andrew Siaw-Asamoah, Anthony Spadaro, Sophia Faude, Christopher K. Snider, M. Kit Delgado, Margaret Lowenstein, Kyle Kampman, Jeanmarie Perrone, Lewis S. Nelson, Austin S. Kilaru

**Affiliations:** 1 Division of General Internal Medicine, Perelman School of Medicine at the University of Pennsylvania, Philadelphia; 2 Center for Addiction Medicine and Policy, Perelman School of Medicine at the University of Pennsylvania, Philadelphia; 3 Department of Medicine, University of Colorado Anschutz Medical Campus, Aurora; 4 Internal Medicine Residency Program, Perelman School of Medicine at the University of Pennsylvania, Philadelphia; 5 Department of Emergency Medicine, Rutgers New Jersey Medical School, Newark; 6 Emergency Medicine Residency, NYU Langone Health, New York, New York; 7 Department of Emergency Medicine, Perelman School of Medicine at the University of Pennsylvania, Philadelphia; 8 Department of Psychiatry, Perelman School of Medicine at the University of Pennsylvania, Philadelphia

## Abstract

**Importance:**

Buprenorphine treatment of opioid use disorder (OUD) is safe and effective, but opioid withdrawal during treatment initiation is associated with poor retention in care. As fentanyl has replaced heroin in the drug supply, case reports and surveys have indicated increased concern for buprenorphine-precipitated withdrawal (PW); however, some observational studies have found a low incidence of PW.

**Objective:**

To estimate buprenorphine PW incidence and assess factors associated with PW among emergency department (ED) or hospitalized patients.

**Design, Setting, and Participants:**

This retrospective cohort study at 3 academic hospitals in Philadelphia, Pennsylvania, included adults with OUD who underwent traditional or high-dose buprenorphine initiation between January 1, 2020, and December 31, 2021. Exclusion criteria included low-dose buprenorphine initiation and missing documentation of opioid withdrawal severity within 4 hours of receiving buprenorphine.

**Exposure:**

Buprenorphine initiation with an initial dose of at least 2 mg of sublingual buprenorphine after a Clinical Opiate Withdrawal Scale (COWS) score of 8 or higher. Additional exposures included 4 predefined factors potentially associated with PW: severity of opioid withdrawal before buprenorphine (COWS score of 8-12 vs ≥13), initial buprenorphine dose (2 vs 4 or ≥8 mg), body mass index (BMI) (<25 vs 25 to <30 or ≥30; calculated as weight in kilograms divided by height in meters squared), and urine fentanyl concentration (0 to <20 vs 20 to <200 or ≥200 ng/mL).

**Main Outcome and Measures:**

The main outcome was PW incidence, defined as a 5-point or greater increase in COWS score from immediately before to within 4 hours after buprenorphine initiation. Logistic regression was used to estimate the odds of PW associated with the 4 aforementioned predefined factors.

**Results:**

The cohort included 226 patients (150 [66.4%] male; mean [SD] age, 38.6 [10.8] years). Overall, 26 patients (11.5%) met criteria for PW. Among patients with PW, median change in COWS score was 9 points (IQR, 6-13 points). Of 123 patients with confirmed fentanyl use, 20 (16.3%) had PW. In unadjusted and adjusted models, BMI of 30 or greater compared with less than 25 (adjusted odds ratio [AOR], 5.12; 95% CI, 1.31-19.92) and urine fentanyl concentration of 200 ng/mL or greater compared with less than 20 ng/mL (AOR, 8.37; 95% CI, 1.60-43.89) were associated with PW.

**Conclusions and Relevance:**

In this retrospective cohort study, 11.5% of patients developed PW after buprenorphine initiation in ED or hospital settings. Future studies should confirm the rate of PW and assess whether bioaccumulated fentanyl is a risk factor for PW.

## Key Points

**Question:**

What is the incidence of buprenorphine-precipitated opioid withdrawal among patients with opioid use disorder (OUD) and fentanyl use who complete traditional or high-dose buprenorphine initiation?

**Findings:**

In this cohort study of 226 adult emergency department or hospitalized patients with opioid withdrawal severity documented within 4 hours of buprenorphine initiation, 12% developed precipitated withdrawal.

**Meaning:**

The findings suggest that a minority of persons using fentanyl are at risk of buprenorphine-precipitated withdrawal when starting OUD treatment and that additional research is needed to identify risk factors for precipitated withdrawal.

## Teaser

This cohort study assesses the incidence of precipitated opioid withdrawal after initiation of buprenorphine among adult emergency department or hospitalized patients with opioid use disorder.

## Introduction

Buprenorphine treatment for opioid use disorder (OUD) is safe,^1^ effective,^2^ and scalable under existing US federal regulations.^3^ Buprenorphine is a partial μ-opioid receptor agonist with high binding affinity; when introduced while a full-agonist opioid such as fentanyl or heroin is present, buprenorphine displaces the full agonist and can precipitate sudden opioid withdrawal.^2^ To prevent this phenomenon of precipitated withdrawal (PW), the traditional initiation approach is to administer successive doses of 2 to 4 mg of sublingual buprenorphine at least 8 to 16 hours after the last use of a short-acting opioid, once patients develop opioid withdrawal (typically assessed as a Clinical Opiate Withdrawal Scale [COWS] score ≥8 on a scale of 0-48, with higher scores indicating more severe opioid withdrawal).^2^ This approach quickly and effectively relieves opioid withdrawal^1,4^ with a low rate of PW among individuals who use short-acting opioids such as heroin or oxycodone.^5^

As fentanyl has adulterated or replaced heroin in the unregulated drug supply and as access to buprenorphine treatment has expanded, patient^6,7,8,9,10^ and clinician^11,12,13^ reports of buprenorphine PW have increased. Patients have described PW despite 48 hours of abstinence from fentanyl,^7^ and case series^11,12,13^ have documented that some patients using fentanyl develop PW despite moderate opioid withdrawal (COWS score ≥13). Since fentanyl is detectable in urine for an average of 7 days after last use for persons with OUD,^14,15^ there is concern that fentanyl retained in adipose or skeletal muscle after chronic use might increase the risk of PW despite 8 to 48 hours of abstinence.^7,16^ Due to fear of PW, some patients using fentanyl now decline buprenorphine,^6,8,9^ and some clinicians use alternative buprenorphine initiation approaches such as low-dose initiation with opioid continuation.^17,18^

Despite patient reports^6,7,8,9,10^ and case series^11,12,13^ that suggest increased concern about PW, 2 recent cohort studies reported a low rate of buprenorphine PW.^19,20^ A secondary analysis of a prospective, multisite emergency department (ED) trial reported that incidence of PW was 0.8%,^19^ while a retrospective cohort study of high-dose initiation (initial doses of ≥8 mg of sublingual buprenorphine) reported PW incidence of 1.6%.^20^ Given the discordant evidence to date, it is essential to understand the incidence of buprenorphine PW in an observed clinical setting.

The primary goal of this study was to estimate the incidence of buprenorphine PW among ED and hospitalized patients receiving traditional or high-dose buprenorphine initiation in a community with high fentanyl prevalence. The secondary goal was to examine whether 4 prespecified factors (severity of opioid withdrawal before buprenorphine, initial dose of buprenorphine, body mass index [BMI; calculated as weight in kilograms divided by height in meters squared], and urine fentanyl concentration) were associated with development of PW.

## Methods

### Study Design and Setting

We conducted a retrospective cohort study of adult patients in Philadelphia, Pennsylvania, who presented for hospital care between January 1, 2020, and December 31, 2021, at 3 urban academic hospitals. During this period, fentanyl was the primary opioid by weight in 86% to 100% of unregulated powdered opioids in Philadelphia.^21,22,23,24^ We extracted detailed encounter-level data from the electronic health record (EHR) through Clarity (Epic Systems Corporation). During the study period, buprenorphine initiation strategies were based on clinician discretion. Clinicians had access to health system guidelines for buprenorphine initiation in hospital settings. Emergency department and inpatient physicians or nurses measured COWS scores before and after buprenorphine initiation through an EHR calculator that recorded the time and individual COWS components. An institutional review board at the University of Pennsylvania approved this study with a waiver of informed consent according to 45 CFR §46. We followed the Strengthening the Reporting of Observational Studies in Epidemiology (STROBE) reporting guideline for cohort studies.^25^

### Study Participants

This study included adults with OUD who received traditional buprenorphine initiation (initial dose of 2-4 mg of sublingual buprenorphine after a COWS score ≥8^2^) or high-dose buprenorphine initiation (initial dose of ≥8 mg of sublingual buprenorphine after a COWS score ≥8^20^) in the ED or hospital. We identified patients with OUD based on (1) an *International Statistical Classification of Diseases, Tenth Revision, Clinical Modification* diagnosis code for the hospital encounter or any encounter in the EHR during the preceding year (codes are listed in eTable 1 in Supplement 1)^16,26^; (2) administration of naloxone in the ED; and/or (3) an ED chief complaint suggestive of OUD or opioid overdose.

We excluded patients who initiated buprenorphine using low-dose initiation with opioid continuation^17,18^ and patients who initiated buprenorphine with subcutaneous extended-release buprenorphine as part of a clinical trial.^27^ We also excluded patients missing COWS documentation within 4 hours before buprenorphine administration and patients with minimal baseline opioid withdrawal (defined as COWS score <8) on the most recent withdrawal assessment prior to receiving buprenorphine. Last, we excluded patients with missing COWS documentation within 4 hours after buprenorphine initiation, precluding assessment of PW. To examine for potential selection bias introduced by these exclusions, we reported characteristics of excluded groups in eTable 2 in Supplement 1.

To estimate PW incidence for persons using fentanyl and to assess urine fentanyl concentration as a factor potentially associated with PW, we defined a secondary cohort consisting of patients who had urine drug testing performed and who had fentanyl or norfentanyl (the major fentanyl metabolite) detected. During the study period, hospital urine drug tests included a rapid point-of-care fentanyl assay; all specimens with fentanyl detected on this rapid assay had confirmatory testing performed using liquid chromatography–mass spectrometry, with an analytical measurement range of 2 to 1000 ng/mL for fentanyl and 5 to 1000 ng/mL for norfentanyl.^16^

Race and ethnicity, ascertained by self-report as documented in the EHR, were included in the analysis because of racial disparities in access to OUD treatment. Categories were Hispanic, non-Hispanic Black (hereafter, *Black*), non-Hispanic White (hereafter, *White*), and other (included American Indian, Asian, and Pacific Islander).

### Primary Outcome

The primary outcome was PW. We followed a prior study to define PW as an increase of 5 points or greater in COWS score between the score immediately preceding the first dose of buprenorphine and the peak COWS score within 4 hours after the first dose of buprenorphine.^19^ We allowed for 4 hours after the first dose of buprenorphine because withdrawal assessments and documentation may be delayed in clinical settings.^28^ To contextualize this outcome, we reported the mean time between COWS assessments used to determine PW and the median increase in COWS score among patients with PW.

### Predefined Factors Potentially Associated With PW

We examined the association of 4 predefined factors potentially associated with PW. This analysis should be considered hypothesis generating. First, we assessed whether patients with a COWS score of 8 to 12 (compared with ≥13) prior to receiving buprenorphine had higher rates of PW based on recent guidelines from the Substance Abuse and Mental Health Services Administration.^29^ Second, we hypothesized that higher initial doses of buprenorphine would be associated with higher rates of PW based on experimental data showing that successive split doses of buprenorphine might be less likely to precipitate withdrawal than a combined single dose.^30^ Third, we hypothesized that higher BMI might be associated with higher rates of PW because fentanyl bioaccumulates in adipose and muscle tissue and higher BMI is associated with slower fentanyl clearance.^15^

Fourth, we hypothesized that higher urine fentanyl concentration may be associated with higher rates of PW because higher urine fentanyl concentrations may be a biomarker of ongoing μ-opioid agonism from fentanyl despite a COWS score of 8 or higher.^16^ Our data reported all urine fentanyl concentrations over 1000 ng/mL as “>1000”; to account for this censoring, we reviewed the distribution of urine fentanyl concentration and stratified this variable into evenly distributed terciles of 0 to less than 20 ng/mL, 20 to less than 200 ng/mL, and 200 or more ng/mL.

### Statistical Analysis

We used descriptive statistics to estimate the incidence of PW and logistic regression to estimate unadjusted and adjusted odds of PW associated with the aforementioned predefined factors. In adjusted models, we controlled for all the predefined covariates. We used Stata, version 16.0 (StataCorp LLC). All statistical tests were 2-sided with a significance level of *P* < .05.

As a sensitivity analysis, we alternatively defined PW as an increase in COWS score of 6 or more points within 1 hour of receiving buprenorphine. An increase in COWS of 6 or more points is clinically meaningful after an opioid antagonist,^31^ and the shorter time between buprenorphine and COWS assessment is less likely to misclassify progressing, undertreated fentanyl withdrawal (eg, if the initial buprenorphine dose is too low) as PW.^13^ As a second sensitivity analysis, we calculated PW incidence assuming all patients missing a COWS score after receiving buprenorphine did not have PW. In addition, we assessed for an overall trend of increased PW frequency across ordered, evenly distributed terciles of opioid withdrawal severity before receiving buprenorphine (COWS score of 8-10, 11-13, and ≥14) and urine fentanyl concentration (0 to <20, 20 to <200, and ≥200 ng/mL) using the Cochran-Armitage test of trend.

## Results

We identified 1577 patients with OUD who received sublingual buprenorphine, of whom 375 (23.8%) received traditional or high-dose initiation (2 [5.3%] received an initial dose of 16 mg) with documented and timely COWS assessment prior to receiving buprenorphine (Figure). Of these 375 patients, 226 (60.3%) were included in the primary cohort because they could be assessed for PW based on COWS documentation within 4 hours of receiving buprenorphine; COWS score after buprenorphine was unavailable for 149 patients (39.7%). There were 170 patients with urine drug testing performed. Of these, 123 patients (72.4%) had fentanyl or norfentanyl detected and were included in the secondary cohort.

**Figure.**
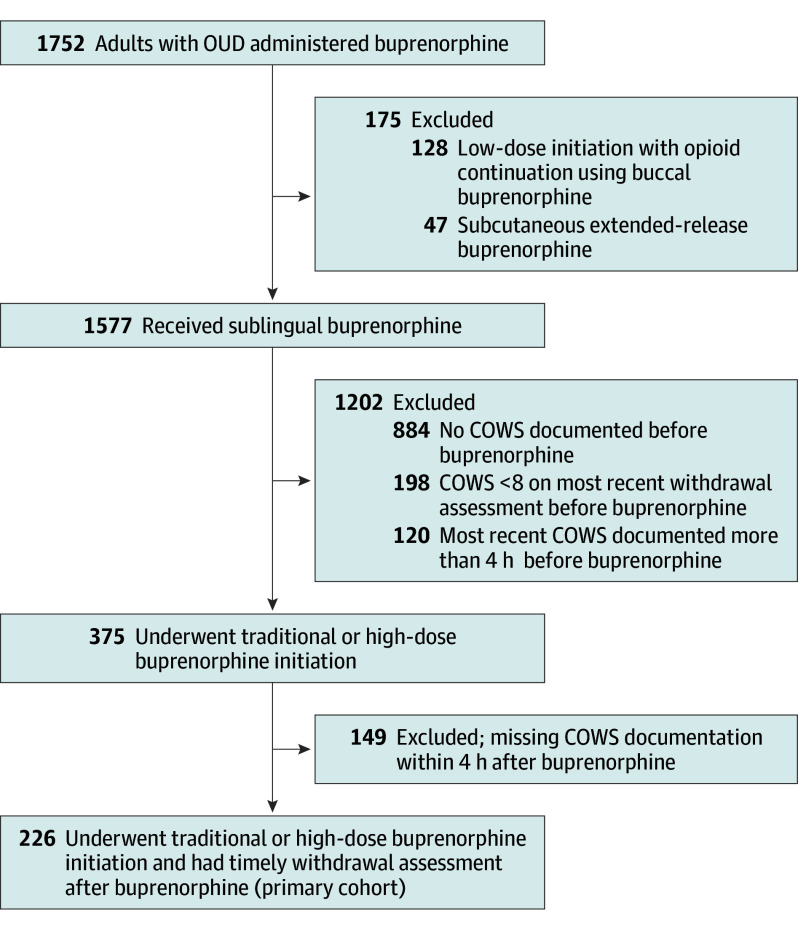
Study Flowchart COWS indicates Clinical Opiate Withdrawal Scale; OUD, opioid use disorder.

For the primary cohort, mean (SD) age was 38.6 (10.8) years; 76 (33.6%) were female, and 150 (66.4%) were male. A total of 72 (31.9%) identified as Black, 17 (7.5%) as Hispanic, 128 (56.6%) as White, and 9 (4.0%) as other. Mean (SD) time between COWS assessments before and after the first dose of buprenorphine was 2.6 (1.2) hours (Table 1). In the secondary cohort, the mean (SD) time from urine drug test specimen collection to buprenorphine initiation was 6.5 (37.3) hours. Compared with all 375 patients who received traditional or high-dose buprenorphine initiation, patients in the primary and secondary cohorts were generally similar but more likely to have a primary diagnosis of infection or overdose, less likely to have a planned discharge from the ED, and more likely to be discharged before medically advised (eTable 2 in Supplement 1).

**Table 1.** Patient Characteristics by Cohort

Characteristic	Patients^a^	
	Primary cohort (n = 226)	Subgroup with confirmed fentanyl use (n = 123)
Age, mean (SD), y	38.6 (10.8)	38.9 (10.3)
Sex		
Female	76 (33.6)	38 (30.9)
Male	150 (66.4)	85 (69.1)
Race and ethnicity		
Hispanic	17 (7.5)	10 (8.1)
Non-Hispanic Black	72 (31.9)	34 (27.6)
Non-Hispanic White	128 (56.6)	74 (60.2)
Other^b^	9 (4.0)	5 (4.1)
Primary insurance		
Medicaid	153 (67.7)	83 (67.5)
Medicare	25 (11.1)	16 (13.0)
Private	39 (17.3)	22 (17.9)
Uninsured	9 (4.0)	2 (1.6)
ED visits in prior 12 mo, No.		
0	95 (42.0)	58 (47.2)
1-3	87 (38.5)	43 (35.0)
≥4	44 (19.5)	22 (17.9)
Hospital admissions in prior 12 mo, No.		
0	169 (74.8)	92 (74.8)
1-3	46 (20.4)	26 (21.1)
≥4	11 (4.9)	5 (4.1)
Charlson Comorbidity Index, mean (SD)	0.9 (1.8)	1.0 (2.1)
Primary diagnosis at discharge^c^		
Related to substance use or withdrawal	98 (43.4)	45 (36.6)
Opioid overdose, other overdose, or intoxication	17 (7.5)	13 (10.6)
Infection or wound	59 (26.1)	37 (30.1)
Other medical	45 (19.9)	25 (20.3)
Other psychiatric	7 (3.1)	3 (2.4)
Discharge disposition		
Planned discharge from ED	58 (25.7)	21 (17.1)
Planned discharge after hospital admission or observation	123 (54.4)	74 (60.2)
Discharged before medically advised	45 (19.9)	28 (22.8)
Drugs detected by urine drug testing^d^		
Fentanyl or norfentanyl	123/170 (72.4)	123 (100)
Methadone	12/170 (7.1)	8 (6.5)
Buprenorphine	10/170 (5.9)	9 (7.3)
Other opioid^e^	75/170 (44.1)	64 (52.0)
Methamphetamine	12/170 (7.1)	11 (8.9)
Cocaine	50/170 (29.4)	45 (36.6)
Benzodiazepines	60/170 (35.3)	42 (34.1)
Setting where buprenorphine was started		
ED	117 (51.8)	56 (45.5)
Hospital admission	109 (48.2)	67 (54.5)
Initial dose of sublingual buprenorphine, mg		
2	31 (13.7)	21 (17.1)
4	145 (64.2)	80 (65.0)
≥8^f^	50 (22.1)	22 (17.9)
Timing of COWS assessments, mean (SD), h		
Between last COWS and buprenorphine	0.9 (0.9)	0.9 (0.8)
Between buprenorphine and peak COWS score within 4 h after buprenorphine	1.6 (0.9)	1.6 (0.9)
Between COWS before and after buprenorphine	2.6 (1.2)	2.5 (1.2)

Abbreviations: COWS, Clinical Opiate Withdrawal Scale; ED, emergency department.

^a^ Data are presented as number (percentage) of participants unless otherwise indicated.

^b^ Included American Indian, Asian, and Pacific Islander.

^c^ Definitions are given in eTable 4 in Supplement 1.

^d^ For the primary cohort, percentage was calculated using the 170 patients with urine drug testing performed as the denominator.

^e^ Includes oxycodone, codeine, hydrocodone, dihydrocodeine, hydromorphone, morphine, acetylmorphine, oxymorphone, and tramadol.

^f^ Two patients received an initial dose of 16 mg of sublingual buprenorphine, neither of whom developed precipitated withdrawal.

### Incidence of PW

Of the 226 patients in the primary cohort, 26 (11.5%) met criteria for PW. Among patients with PW, median change in COWS score was 9 points (IQR, 6-13 points), with a mean (SD) of 2.0 (1.1) hours between COWS assessments before and after buprenorphine initiation. Detailed data on each PW case are reported in eTable 3 in Supplement 1. Of 123 patients in the fentanyl or norfentanyl subgroup, 20 (16.3%) developed PW. In sensitivity analyses, PW incidence was 9 of 47 patients (19.1%) using the stricter definition of PW (≥6-point increase in COWS score within 1 hour of buprenorphine) and PW incidence was 26 of 365 patients (7.1%) if all patients missing COWS documentation after buprenorphine were assumed not to have had PW.

### Factors Associated With PW

For the primary cohort of all patients with traditional or high-dose buprenorphine initiation, logistic regression models showed no significant unadjusted or adjusted associations between PW and the severity of opioid withdrawal before buprenorphine, the initial dose of buprenorphine, or BMI (Table 2). In the secondary cohort of patients with fentanyl or norfentanyl detected on urine drug testing, there were also no significant unadjusted or adjusted associations between PW and severity of opioid withdrawal before buprenorphine or initial dose of buprenorphine (Table 3).

**Table 2.** Factors Associated With PW in the Primary Cohort

Factor	Patients, No. (%)			PW incidence, No./total No. (%)	Unadjusted OR (95% CI)^a^	*P* value^b^	Adjusted OR (95% CI)^a^	*P* value^b^
	All (n = 226)	PW (n = 26)	No PW (n = 200)					
Initial dose of SL buprenorphine, mg								
2	31 (13.7)	2 (7.7)	29 (14.5)	2/31 (6.5)	1 [Reference]	.42	1 [Reference]	.44
4	145 (64.2)	16 (61.5)	129 (64.5)	16/145 (11.0)	1.79 (0.39-8.26)		1.90 (0.41-8.87)	
≥8	50 (22.1)	8 (30.8)	42 (21.0)	8/50 (16.0)	2.76 (0.55-8.26)		2.80 (0.55-14.31)	
Opioid withdrawal severity before buprenorphine, COWS score^c^								
8-12	144 (63.7)	15 (57.7)	129 (64.5)	15/144 (10.4)	1 [Reference]	.50	1 [Reference]	.54
≥13	82 (36.3)	11 (42.3)	71 (35.5)	11/82 (13.4)	1.33 (0.58-3.06)		1.30 (0.56-3.04)	
BMI category								
<25	121 (53.5)	12 (46.2)	98 (49.0)	12/121 (9.9)	1 [Reference]	.39	1 [Reference]	.38
25 to <30	73 (32.3)	8 (30.8)	65 (32.5)	8/73 (11.0)	1.12 (0.45-2.88)		1.06 (0.41-2.76)	
≥30	32 (14.2)	6 (23.1)	26 (13.0)	6/32 (18.8)	2.10 (0.72-6.11)		2.09 (0.71-6.15)	

Abbreviations: BMI, body mass index (calculated as weight in kilograms divided by height in meters squared); COWS, Clinical Opiate Withdrawal Scale; OR, odds ratio; PW, precipitated withdrawal; SL, sublingual.

^a^ Using logistic regression. Adjusted models controlled for all factors as covariates.

^b^ Overall test for any association using logistic regression.

^c^ Score range 0 to 48, with higher scores indicating more severe opioid withdrawal.

**Table 3.** Factors Associated With PW Among Patients With Confirmed Fentanyl Use

Factor	Patients, No. (%)			PW incidence, No./total No. (%)	Unadjusted OR (95% CI)^a^	*P* value^b^	Adjusted OR (95% CI)^a^	*P* value^b^
	All (n = 123)	PW (n = 20)	No PW (n = 103)					
Initial dose of SL buprenorphine, mg								
2	21 (17.1)	2 (10.0)	19 (18.4)	2/21 (9.5)	1 [Reference]	.51	1 [Reference]	.55
4	80 (65.0)	13 (65.0)	67 (65.0)	13/80 (16.3)	1.84 (0.38-8.89)		1.77 (0.31-10.06)	
≥8	22 (17.9)	5 (25.0)	17 (16.5)	5/22 (22.7)	2.79 (0.48-16.33)		2.87 (0.41-20.00)	
Opioid withdrawal severity before buprenorphine, COWS score^c^								
8-12	81 (65.6)	12 (60.0)	69 (67.0)	12/81 (14.8)	1 [Reference]	.55	1 [Reference]	.29
≥13	42 (34.1)	8 (40.0)	34 (33.0)	8/42 (19.0)	1.35 (0.51-3.62)		1.81 (0.60-5.44)	
BMI category								
<25	67 (54.5)	7 (35.0)	52 (50.5)	7/67 (10.4)	1 [Reference]	.07	1 [Reference]	.06
25 to <30	38 (30.9)	7 (35.0)	31 (30.1)	7/38 (18.4)	1.93 (0.62-6.01)		2.01 (0.60-6.70)	
≥30	18 (14.6)	6 (30.0)	12 (11.7)	6/18 (33.3)	4.29 (1.22-15.02)		5.12 (1.31-19.92)	
Urine fentanyl concentration, ng/mL								
0 to <20	33 (26.8)	2 (10.0)	31 (30.1)	2/33 (6.1)	1 [Reference]	.02	1 [Reference]	.02
20 to <200	50 (40.7)	6 (30.0)	44 (42.7)	6/50 (12.0)	2.59 (0.69-9.68)		2.61 (0.47-14.57)	
≥200	40 (32.5)	12 (60.0)	28 (27.2)	12/40 (30.0)	8.14 (2.42-27.35)		8.37 (1.60-43.89)	

Abbreviations: BMI, body mass index (calculated as weight in kilograms divided by height in meters squared); COWS, Clinical Opiate Withdrawal Scale; OR, odds ratio; PW, precipitated withdrawal; SL, sublingual.

^a^ Using logistic regression. Adjusted models controlled for all factors as covariates.

^b^ Overall test for any association using logistic regression.

^c^ Score range 0 to 48, with higher scores indicating more severe opioid withdrawal.

However, in the secondary cohort, BMI of 30 or greater compared with BMI less than 25 was associated with PW in unadjusted models (odds ratio [OR], 4.29; 95% CI, 1.22-15.02) and fully adjusted models (adjusted OR [AOR], 5.12; 95% CI, 1.31-19.92). High fentanyl concentration (≥200 ng/mL) compared with low concentration (0 to <20 ng/mL) was also associated with PW in unadjusted models (OR, 8.14; 95% CI, 2.42-27.35) and fully adjusted models (AOR, 8.37; 95% CI, 1.60-43.89). Across terciles of opioid withdrawal severity before buprenorphine, there was no linear trend for higher PW incidence with lower COWS score (slope [SE], 0.01 [0.04]; *P* = .79) (Table 4). In contrast, a linear trend for higher PW incidence was detected with higher urine fentanyl concentration (slope [SE], 0.12 [0.04]; *P* = .005).

**Table 4.** Incidence of PW by Urine Fentanyl Concentration and Opioid Withdrawal Severity Before Buprenorphine Initiation

Withdrawal severity, COWS score^a^	PW incidence, No./total No. (%)			
	Urine fentanyl concentration, ng/mL^b^			Total^a^
	0-19	20-199	≥200	
8-10	0/12	4/23 (17.4)	5/21 (23.8)	9/56 (16.1)
11-13	0/9	1/16 (6.3)	4/10 (40.0)	5/35 (14.3)
≥14	2/12 (16.7)	1/11 (9.1)	3/9 (33.3)	6/32 (18.8)
Total^b^	2/33 (6.1)	6/50 (12.0)	12/40 (30.0)	NA

Abbreviations: COWS, Clinical Opiate Withdrawal Scale; NA, not applicable; PW, precipitated withdrawal.

^a^ *P* = .79 for Cochran-Armitage test of linear trend of PW risk by increasing COWS score (range 0-48, with higher scores indicating more severe opioid withdrawal).

^b^ *P* = .004 for Cochran-Armitage test of linear trend of PW risk by increasing urine fentanyl concentration.

## Discussion

In this retrospective cohort study of ED and hospitalized patients with OUD, 11.5% of patients developed PW while starting buprenorphine with traditional or high-dose initiation. Among patients with confirmed fentanyl use, 16.3% developed PW. Exploratory analyses of predefined factors found that high BMI (≥30) and high urine fentanyl concentration (≥200 ng/mL) were associated with PW among patients using fentanyl, whereas there was no detected association of initial opioid withdrawal severity or initial buprenorphine dose with PW.

In this ED- and hospital-based study in a community with high fentanyl prevalence, PW incidence was higher than estimates from a study of clinical trial data^19^ and prior ED-based studies^20^ but lower than estimates based on patient report.^7^ We used the same definition of PW as the largest study to date on PW incidence, a secondary analysis of a randomized clinical trial that found an incidence of 1%,^19^ but the clinical sample in our study might be more representative of the general population due to selection bias inherent in clinical trial enrollment.^32^ Our estimate of PW incidence could have been biased by the 39.7% of patients who were missing COWS documentation after buprenorphine initiation. These missing data may have led to an overestimate of PW incidence if patients with PW were more likely to have had withdrawal documented. To account for this, we conducted a sensitivity analysis assuming all patients with missing COWS scores after receiving buprenorphine did not have PW and found a lower bound of 7.1% PW incidence, which is still substantially higher than in prior studies.^19,20^ On the other hand, these missing data may have led to underestimates of PW incidence if patients with PW declined formal withdrawal assessments or left the hospital without having withdrawal documented. Notably, PW incidence in this study was lower than in a survey of patients entering addiction treatment facilities in which 37% of 685 respondents using fentanyl reported severe opioid withdrawal after taking buprenorphine.^7^

It is important to establish the incidence of buprenorphine PW from fentanyl because fentanyl is the predominant opioid involved in overdoses^33^ and because buprenorphine is the only US Food and Drug Administration–approved medication for OUD that is both associated with decreased mortality^34^ and available across all US treatment settings.^1,2^ Opioid withdrawal during treatment initiation is associated with poor retention in outpatient care^35^ and with hospital discharges before medically advised.^36,37^ Concern for PW has led some individuals to decline treatment^6,8^ and prompted 2 unions representing people who use drugs to call for new ways of initiating buprenorphine.^9^ Precipitated withdrawal may also dissuade clinicians, especially those less experienced with addiction treatment,^38^ from offering buprenorphine. This study found that PW occurred in only a minority of cases, suggesting that traditional or high-dose buprenorphine initiation should still be successful for most individuals using fentanyl. However, the higher incidence of PW in this study compared with prior research^19,20^ indicates that further study is needed to identify at-risk individuals and elucidate underlying mechanisms.

To our knowledge, our exploratory analysis of factors associated with PW provides the first evidence supporting the theory that persistent opioid agonism from bioaccumulated fentanyl contributes to PW in a minority of persons using fentanyl.^16^ With chronic use, fentanyl accumulates in muscle and fat.^39^ As a result, terminal kidney clearance of fentanyl takes an average of 7 days^14^ for individuals with OUD and may be slower for people with higher BMI.^15^ We found that higher BMI and higher urine fentanyl concentration were associated with PW despite a COWS score of 8 or higher prior to receiving buprenorphine. One potential interpretation of these findings is that during early opioid abstinence, some individuals have low enough μ-opioid receptor activation to experience clinical opioid withdrawal but high enough μ-opioid receptor activation from bioaccumulated fentanyl to put them at risk of buprenorphine PW. Based on this interpretation, the amount of fentanyl exposure over time, the route of administration, and each person’s capacity to store fentanyl might determine PW risk, not simply the presence of any fentanyl in the drug supply. These factors might explain divergent experiences with PW across regions. Currently in Philadelphia, 99% of powdered opioids contain fentanyl, intravenous fentanyl use is common, and average fentanyl purity (percentage of fentanyl by weight) is 12% to 15% but can be as high as 40%.^40^ In other regions, heroin is still available,^33^ fentanyl is smoked more than injected,^41^ and fentanyl purity may be lower.^42^

If confirmed in future studies, the findings of this study could help clinicians estimate risk of PW.^43^ Clinical applications of quantitative urine fentanyl tests are currently limited by the time and cost required for mass spectrometry. However, existing point-of-care urine fentanyl lateral flow assays (“dipsticks”) are cheap, take minutes to complete, and have a limit of detection of 20 ng/mL in urine.^44^ These tests might be adapted to create a rapid, low-cost, point-of-care test to inform PW risk assessments.

This study did not detect an association between severity of prebuprenorphine opioid withdrawal and PW. Future studies with larger sample sizes should assess whether a higher COWS score before receiving buprenorphine reduces the risk of PW, as suggested by newer clinical guidelines for traditional or high-dose buprenorphine initiations.^17,29^ Additionally, this study did not detect an association between initial buprenorphine dose and PW. Incidence of PW was descriptively higher among individuals who received higher initial doses of buprenorphine, making it less likely that our PW definition misclassified undertreated withdrawal as PW. Only 2 patients received an initial dose of 16 mg of sublingual buprenorphine (neither of whom developed PW); thus, it is possible that response to buprenorphine doses of 16 mg or greater differ from responses to 8 mg.^45^

### Limitations

This study has several limitations. First, we used a retrospective cohort design with clinical data; this may introduce unobserved bias and confounding. Second, our sample size limited statistical power to detect small-magnitude associations between predefined factors and PW. Third, there was a high rate of missingness in documented COWS assessments both before and after buprenorphine administration. Fourth, we could not distinguish patients newly initiating buprenorphine from those continuing prior treatment, although the inclusion of patients continuing treatment would lead to underestimation of PW incidence for buprenorphine inductions. We examined the potential effects of these limitations by comparing observed characteristics of excluded patients with those of patients included in the final cohort and by reporting PW incidences across various cohorts and definitions of PW as sensitivity analyses.

Additionally, our data did not allow us to normalize fentanyl concentrations for urine dilution,^16,46^ and fentanyl itself might not be implicated in PW if higher urine fentanyl concentration is just a proxy for higher amount or frequency of opioid use. Future research should confirm the association of urine fentanyl concentration with PW while accounting for urine dilution. Finally, the focus of this study on patients in the ED or hospital may have limited generalizability to patients initiating buprenorphine at home; these patients might have less risk for PW given less severe co-occurring illness but potentially greater risk without direct clinical supervision.

## Conclusions

In this cohort study, PW occurred in 11.5% of hospital-based traditional or high-dose buprenorphine initiations and was more frequent among patients with confirmed fentanyl use. As fentanyl adulteration increases in the drug supply and as access to buprenorphine expands, future research should confirm the rate of buprenorphine PW, assess whether bioaccumulated fentanyl is responsible for PW, and determine whether BMI and urine fentanyl concentration can help clinicians optimize buprenorphine initiation for individual patients.

## Notes

Supplement 1. **eTable 1.** *ICD-10-CM* Diagnosis Codes for Opioid Use Disorder **eTable 2.** Patient Characteristics of Excluded Cohorts **eTable 3.** Precipitated Withdrawal Case Descriptions **eTable 4.** Discharge Diagnosis Categories

Supplement 2.
Data Sharing Statement
