# Supplementary material for: Buprenorphine-Precipitated Withdrawal Among Hospitalized Patients Using Fentanyl
Source: JAMA Netw Open. 2024 Sep 27;7(9):e2435895. doi: 10.1001/jamanetworkopen.2024.35895 (PMC11437388; doi:10.1001/jamanetworkopen.2024.35895)
Supplement: Supplement 1. — eTable 1. ICD-10-CM Diagnosis Codes for Opioid Use Disorder eTable 2. Patient Characteristics of Excluded Cohorts eTable 3. Precipitated Withdrawal Case Descriptions eTable 4. Discharge Diagnosis Categories [file jamanetwopen-e2435895-s001.pdf]

Supplementary Online Content

Thakrar AP, Christine PJ, Siaw-Asamoah A, et al. Buprenorphine-precipitated withdrawal among hospitalized patients using fentanyl. *JAMA Netw Open*. 2024;7(10):e2435895. doi:10.1001/jamanetworkopen.2024.35895

eTable 1. *ICD-10-CM* Diagnosis Codes for Opioid Use Disorder

eTable 2. Patient Characteristics of Excluded Cohorts

eTable 3. Precipitated Withdrawal Case Descriptions

eTable 4. Discharge Diagnosis Categories

This supplementary material has been provided by the authors to give readers additional information about their work.

© 2024 Thakrar AP et al. *JAMA Network Open*.

eTable 1. *ICD-10-CM* diagnosis codes for opioid use disorder

ICD-10-CM Code	Description
F11.1	Opioid use disorder, mild
F11.10	Opioid use disorder, mild
F11.12	Opioid use disorder, mild with intoxication
F11.14	Opioid use disorder, mild with opioid-induced mood disorder
F11.15	Opioid use disorder, mild with opioid-induced psychotic disorder
F11.18	Opioid use disorder, mild with other opioid-induced disorder
F11.19	Opioid use disorder, mild with unspecified opioid-induced disorder
F11.2	Opioid use disorder, moderate or severe
F11.22	Opioid use disorder, moderate or severe with intoxication
F11.23	Opioid use disorder, moderate or severe with withdrawal
F11.24	Opioid use disorder, moderate or severe with opioid-induced mood disorder
F11.25	Opioid use disorder, moderate or severe with opioid-induced psychotic disorder
F11.28	Opioid-dependence with other opioid-induced disorder
F11.29	Opioid use disorder, moderate or severe with unspecified opioid-induced disorder
F11.9	Opioid use, unspecified
F11.90	Opioid use, uncomplicated
F11.92	Opioid use, unspecified with intoxication
F11.93	Opioid use, unspecified with withdrawal
F11.94	Opioid use, unspecified with opioid-induced mood disorder
F11.95	Opioid use, unspecified with opioid-induced psychotic disorder
F11.98	Opioid use, unspecified with other specified opioid-induced disorder
F11.99	Opioid use, unspecified with unspecified opioid-induced disorder
T40.0	Poisoning by, adverse effect of and underdosing of opium
T40.1	Poisoning by and adverse effect of heroin
T40.2	Poisoning by, adverse effect of and underdosing of other opioids
T40.3	Poisoning by, adverse effect of and underdosing of methadone
T40.4	Poisoning by, adverse effect of and underdosing of other synthetic narcotics
T40.6	Poisoning by, adverse effect of and underdosing of other and unspecified narcotics

© 2024 Thakrar AP et al. *JAMA Network Open*.

eTable 2. Patient characteristics of excluded cohorts

	No. (%)						
	**Adults with OUD administered sublingual buprenorphine**	**Missing COWS documentation before first buprenorphine administration**	**COWS < 8 before first buprenorphine administration**	**All traditional buprenorphine initiations**	**Missing COWS documentation <4h after first buprenorphine administration**	**Primary cohort**	**Secondary cohort**
		Excluded cohort	*Excluded cohort*		*Excluded cohort*		
	**(n = 1577)**	**(n = 884)**	**(n = 198)**	**(n = 375)**	**(n = 149)**	**(n = 226)**	**(n=123)**
Age, mean (SD)	43.0(13.2)	44.6(14.0)	40.9(12.3)	39.9(11.2)	41.8(11.7)	38.6(10.8)	38.9(10.3)
Female	524 (33.2)	283 (32.0)	53 (26.8)	144 (38.4)	68 (45.6)	76 (33.6)	38 (30.9)
Race/Ethnicity							
Black (non-Hispanic)	541 (34.3)	297 (33.6)	78 (39.4)	130 (34.7)	58 (38.9)	72 (31.9)	34 (27.6)
White (non-Hispanic)	888 (56.3)	521 (58.9)	94 (47.5)	201 (53.6)	73 (49.0)	128 (56.6)	74 (60.2)
Hispanic	91 (5.8)	42 (4.8)	13 (6.6)	29 (7.7)	12 (8.1)	17 (7.5)	10 (8.1)
Other	57 (3.6)	24 (2.7)	13 (6.6)	15 (4.0)	6 (4.0)	9 (4.0)	5 (4.1)
Primary insurance							
Medicaid	963 (61.1)	496 (56.1)	146 (73.7)	253 (67.5)	100 (67.1)	153 (67.7)	83 (67.5)
Medicare	216 (13.7)	139 (15.7)	20 (10.1)	42 (11.2)	17 (11.4)	25 (11.1)	16 (13.0)
Private	350 (22.2)	227 (25.7)	28 (14.1)	61 (16.3)	22 (14.8)	39 (17.3)	22 (17.9)
Uninsured	48 (3.0)	22 (2.5)	4 (2.0)	19 (5.1)	10 (6.7)	9 (4.0)	2 (1.6)
ED visits in prior 12 mo							
0	528 (33.5)	272 (30.8)	56 (28.3)	156 (41.6)	61 (40.9)	95 (42.0)	58 (47.2)
1-3	565 (35.8)	305 (34.5)	77 (38.9)	136 (36.3)	49 (32.9)	87 (38.5)	43 (35.0)
≥4	484 (30.7)	307 (34.7)	65 (32.8)	83 (22.1)	39 (26.2)	44 (19.5)	22 (17.9)
Hospital admissions in prior 12 mo							
0	1,040 (65.9)	548 (62.0)	132 (66.7)	280 (74.7)	111 (74.5)	169 (74.8)	92 (74.8)
1-3	412 (26.1)	239 (27.0)	55 (27.8)	81 (21.6)	35 (23.5)	46 (20.4)	26 (21.1)
≥4	125 (7.9)	97 (11.0)	11 (5.6)	14 (3.7)	3 (2.0)	11 (4.9)	5 (4.1)
Charlson Comorbidity Index, mean (SD)	1.7(2.7)	2.1(2.9)	1.5(2.5)	1.0(1.9)	1.2(2.0)	0.9(1.8)	1.0(2.1)

© 2024 Thakrar AP et al. *JAMA Network Open*.

Primary diagnosis on discharge*							
Related to substance use or withdrawal	445 (28.2)	163 (18.4)	83 (41.9)	169 (45.1)	71 (47.7)	98 (43.4)	45 (36.6)
Opioid overdose, other overdose, or intoxication	76 (4.8)	38 (4.3)	8 (4.0)	22 (5.9)	5 (3.4)	17 (7.5)	13 (10.6)
Infection or wound	283 (17.9)	133 (15.0)	39 (19.7)	80 (21.3)	21 (14.1)	59 (26.1)	37 (30.1)
Other medical	695 (44.1)	504 (57.0)	58 (29.3)	89 (23.7)	44 (29.5)	45 (19.9)	25 (20.3)
Other psychiatric	78 (4.9)	46 (5.2)	10 (5.1)	15 (4.0)	8 (5.4)	7 (3.1)	3 (2.4)
Discharge Disposition							
Planned discharge from ED	513 (32.5)	283 (32.0)	82 (41.4)	129 (34.4)	71 (47.7)	58 (25.7)	21 (17.1)
Planned discharge after hospital admission or observation	901 (57.1)	537 (60.7)	96 (48.5)	184 (49.1)	61 (40.9)	123 (54.4)	74 (60.2)
Discharge before medically advised	161 (10.2)	63 (7.1)	19 (9.6)	62 (16.5)	17 (11.4)	45 (19.9)	28 (22.8)
Deceased	2 (0.1)	1 (0.1)	1 (0.5)	0 (0.0)	0 (0.0)	0 (0.0)	0 (0.0)
Setting where buprenorphine was started							
ED	821 (52.1)	464 (52.5)	109 (55.1)	217 (57.9)	100 (67.1)	117 (51.8)	56 (45.5)
Hospital admission	756 (47.9)	420 (47.5)	89 (44.9)	158 (42.1)	49 (32.9)	109 (48.2)	67 (54.5)

* See Appendix Table A3 for definition

© 2024 Thakrar AP et al. *JAMA Network Open*.

eTable 3. Precipitated withdrawal case descriptions

	PATIENT CHARACTERISTICS					URINE DRUG TESTING			BUP. INITIATION		COWS ASSESSMENTS			HOSPITAL DISCHARGE	
**CASE**	**Quarter**	**Age**	**Sex**	**BMI**	**ED Chief Complaint**	**Hours to UDT***	**Drugs detected**	**Fentanyl; norfentanyl concentration (ng/mL)**	**Hours to bup.****	**Initial bup. dose**	**COWS scores, before & after bup.**	**Change in COWS**	**Hours between COWS**	**Hospital LOS, days**	**Discharge plan from hospital**
1	Q1 2020	40s	Female	20.6	Fever	2.2	Opiates	0; 0	40.2	4mg	9; 19	10	2.5	3	BMA
2	Q1 2020	30s	Male	25.0	Withdrawal	No UDT performed			0.6	8mg	14; 27	13	1.6	<1	BMA
3	Q2 2020	30s	Female	18.6	Eye problem	8.5	Fentanyl, benzodiazepines, cocaine	545; >1000	11.0	4mg	25; 30	5	1.1	2	BMA
4	Q2 2020	20s	Female	20.5	Diarrhea	0.6	Amphetamine	0	8.2	4mg	10; 15	5	1.7	9	Transfer
5	Q2 2020	30s	Male	25.9	Withdrawal	9.8	Fentanyl, barbiturates, hydromorphone, morphine	214; >1000	38.3	4mg	15; 28	13	0.4	6	Planned home
6	Q3 2020	60s	Male	28.0	Shortness of breath	20.5	Fentanyl	80; >1000	22.0	4mg	9; 20	11	1.8	5	Planned home
7	Q3 2020	40s	Male	28.8	Stroke alert	5.9	Fentanyl, alcohol, amphetamines, cocaine	49; 82	3.2	4mg	10; 19	9	1.0	4	Planned home
8	Q3 2020	30s	Male	27.5	Rash	27.9	Fentanyl, benzodiazepines, cocaine, methamphetamine, morphine	11; 381	24.5	4mg	18; 32	14	0.9	5	Planned home
9	Q4 2020	50s	Female	22.6	Addiction problem	0.8	Fentanyl	332; >1000	1.1	8mg	9; 14	5	2.2	<1	Planned home
10	Q1 2021	20s	Female	18.8	Detox	3.7	Fentanyl	85; >1000	3.7	2mg	10; 16	6	2.4	<1	Planned home
11	Q1 2021	30s	Male	26.6	Cough	24.2	Fentanyl, benzodiazepines, cocaine	209; >1000	32.3	4mg	11; 29	18	1.3	1	Planned home
12	Q1 2021	50s	Male	26.6	Abdominal pain	7.8	Fentanyl	4; 206	5.5	4mg	23; 32	9	2.9	<1	Planned home
13	Q1 2021	20s	Male	23.7	Abscess	No UDT performed			13.5	8mg	9; 29	20	1.7	1	BMA
14	Q1 2021	30s	Female	28.5	Seizures	2.7	Fentanyl, benzodiazepines, cocaine, morphine, THC, tramadol	252; 999	16.2	4mg	11; 16	5	2.4	1	BMA
15	Q2 2021	50s	Male	23.2	Shortness of breath	9.5	Fentanyl	234; >1000	12.8	4mg	10; 17	7	0.8	1	BMA
16	Q2 2021	30s	Female	34.3	Detox	5.7	Fentanyl, cocaine	393; >1000	22.1	4mg	12; 18	6	3.0	7	Transfer
17	Q2 2021	30s	Male	20.7	Withdrawal	No UDT performed			2.9	8mg	13; 21	8	2.6	<1	Planned home
18	Q2 2021	30s	Female	32.4	Altered mental status	15.7	Fentanyl, benzodiazepines, buprenorphine, methadone, morphine, oxycodone	733; >1000	7.6	4mg	8; 31	23	5.9	4	Planned home

© 2024 Thakrar AP et al. *JAMA Network Open*.

19	Q3 2021	40s	Male	30.5	Wrist pain	14.1	Fentanyl, benzodiazepines, buprenorphine, cocaine, codeine, hydromorphone, morphine, THC	562; >1000	62.0	2mg	13; 21	8	2.9	2	BMA
20	Q3 2021	30s	Male	37.8	Addiction problem	0.1	Fentanyl, amphetamines, benzodiazepines	>1000; >1000	1.3	8mg	10; 19	9	0.6	1	Planned home
21	Q3 2021	40s	Male	22.0	Wound check	6.9	Fentanyl	47; >1000	6.3	8mg	13; 18	5	2.7	8	Planned home
22	Q3 2021	20s	Female	23.8	Vomiting	17.0	Fentanyl, benzodiazepines, cocaine	382; >1000	1.7	4mg	24; 31	7	1.4	6	Transfer
23	Q3 2021	30s	Male	21.3	Wound infection	No UDT performed			2.4	4mg	16; 22	6	1.4	1	BMA
24	Q3 2021	30s	Female	19.2	Wound infection	12.7	Fentanyl, benzodiazepines, buprenorphine, cocaine, oxycodone	146; 363	5.6	8mg	8; 21	13	1.2	1	BMA
25	Q4 2021	30s	Female	30.7	Abdominal pain	0.3	Fentanyl, codeine, morphine, tramadol	428; >1000	8.4	8mg	9; 21	12	2.2	9	Transfer
26	Q4 2021	40s	Male	[Missing]	Withdrawal	1.0	Fentanyl, barbiturates, cocaine	53; >1000	1.1	4mg	19; 34	15	2.5	8	Incarceration

Abbreviations: BMI, Body Mass Index; ED, Emergency Department; COWS, Clinical Opiate Withdrawal Scale; LOS, Length Of Stay; BMA, Before Medically Advised; Bup., Buprenorphine; Fent., Fentanyl; Norfent., Norfentanyl

* Hours from ED presentation to urine specimen collection
** Hours from ED presentation to first dose of buprenorphine

© 2024 Thakrar AP et al. *JAMA Network Open*.

eTable 4. Discharge diagnosis categories

Diagnosis category	Primary discharge diagnosis term
Related to substance use or withdrawal	" use ", " use", "abuse", "withdrawal", "use disorder", "dependence", "drug", "misuse", "addiction", "maintenance"
Opioid overdose, other overdose, or intoxication	"overdose", "intoxication", “poison”
Infection or Wound	"infection", "cellulitis" , "wound" , "abscess" , "pneumonia" , "viral" , "osteomyelitis" , "septic" , "sepsis" , "infectious" , "COVID", "necrotic" , "gangren" , "influenza", "Bacteremia", "blood culture", "externa", "endocarditis", "meningitis", "sinusitis", "pyelonephritis", "laceration"
Other Psychiatric	"suicid" , "depress" , "bipolar" , "schizo" , "anxiety" , "agitation" , "behavior" , "psychotic", "psychosis", "self-harm"
Other Medical	Other

© 2024 Thakrar AP et al. *JAMA Network Open*.
