# Supplementary material for: Buprenorphine-Precipitated Withdrawal Among Hospitalized Patients Using Fentanyl
Source: JAMA Netw Open. 2024 Sep 27;7(9):e2435895. doi: 10.1001/jamanetworkopen.2024.35895 (PMC11437388; doi:10.1001/jamanetworkopen.2024.35895)
Supplement: Supplement 2. — Data Sharing Statement [file jamanetwopen-e2435895-s002.pdf]

Data Sharing Statement

Thakrar. Buprenorphine-Precipitated Withdrawal Among Hospitalized Patients Using Fentanyl. *JAMA Netw Open*. Published October 02, 2024. doi:10.1001/jamanetworkopen.2024.35895

Data
Data available: No
